# Maternal multimorbidity during pregnancy and after childbirth in women in low- and middle-income countries: a systematic literature review

**DOI:** 10.1186/s12884-020-03303-1

**Published:** 2020-10-20

**Authors:** Mary McCauley, Shamsa Zafar, Nynke van den Broek

**Affiliations:** 1 Centre for Maternal and Newborn Health, Liverpool School of Tropical Medicine, Pembroke Place, Liverpool, L3 5QA UK; 2 Fazaia Medical College, Air University, Islamabad, Pakistan

**Keywords:** Maternal morbidity, Multimorbidity, Pregnancy and childbirth, Burden of disease, Measurement, Data collection, Low- and middle-income countries

## Abstract

**Background:**

For every maternal death, 20 to 30 women are estimated to have morbidities related to pregnancy or childbirth. Much of this burden of disease is in women in low- and middle-income countries. Maternal multimorbidity can include physical, psychological and social ill-health. Limited data exist about the associations between these morbidities. In order to address all health needs that women may have when attending for maternity care, it is important to be able to identify all types of morbidities and understand how each morbidity influences other aspects of women’s health and wellbeing during pregnancy and after childbirth.

**Methods:**

We systematically reviewed published literature in English, describing measurement of two or more types of maternal morbidity and/or associations between morbidities during pregnancy or after childbirth for women in low- and middle-income countries. CINAHL plus, Global Health, Medline and Web of Science databases were searched from 2007 to 2018. Outcomes were descriptions, occurrence of all maternal morbidities and associations between these morbidities. Narrative analysis was conducted.

**Results:**

Included were 38 papers reporting about 36 studies (71,229 women; 60,911 during pregnancy and 10,318 after childbirth in 17 countries). Most studies (26/36) were cross-sectional surveys. Self-reported physical ill-health was documented in 26 studies, but no standardised data collection tools were used. In total, physical morbidities were included in 28 studies, psychological morbidities in 32 studies and social morbidities in 27 studies with three studies assessing associations between all three types of morbidity and 30 studies assessing associations between two types of morbidity. In four studies, clinical examination and/or basic laboratory investigations were also conducted. Associations between physical and psychological morbidities were reported in four studies and between psychological and social morbidities in six. Domestic violence increased risks of physical ill-health in two studies.

**Conclusions:**

There is a lack of standardised, comprehensive and routine measurements and tools to assess the burden of maternal multimorbidity in women during pregnancy and after childbirth. Emerging data suggest significant associations between the different types of morbidity.

**Systematic review registration number:**

PROSPERO CRD42018079526.

## Background

Maternal multimorbidities affect millions of women during pregnancy and after childbirth and the burden of ill-health is expected to be highest in women in low-and middle-income countries (LMIC) [1]. For every maternal death, 20 to 30 women have morbidities related to pregnancy or childbirth [2, 3]. More recent studies using new and comprehensive assessment tools suggest that the magnitude of maternal multimorbidity is much larger than previously estimated [4–6]. International targets and the Sustainable Development Goals have a new focus; in addition to preventing maternal mortality, improving health and well-being, as well as “survive and thrive” are the new goals [7]. There is international agreement that all women have the right to the highest attainable standard of health and well-being, also during pregnancy and after childbirth [7, 8]. Estimates of morbidity have until now largely focused on acute and/or severe complications such as haemorrhage, sepsis and eclampsia [9]. The current definition of health is “a state of complete physical, mental, and social well-being and not merely the absence of disease or infirmity” [10]. There are arguments that this definition needs to be re-formulated to consider health in a context of functionality, capacity, adaptability and the ability to perform activities of daily living despite having an illness or disability; but with a continued emphasis on the importance of the three domains of health: physical, psychological and social [11]. There is also debate that current definitions, measurements and timeframes for “multimorbidity”, “co-morbidity”, “morbidity burden” and related constructs are not well conceptualized [11, 12].

Regarding maternal morbidity, a suggested definition is “any health condition attributed to and/or aggravated by pregnancy and childbirth that has a negative impact on women’s well-being” [13]. In order to address all health needs that women may have when attending for maternity care, it is important to be able to identify all types of morbidities and understand how each morbidity influences other aspects of women’s health and wellbeing during pregnancy and after childbirth. To date, lack of data exist regarding measurement and burden of disease described as “maternal morbidity”, “maternal multimorbidity”, or “maternal co-morbidity”; these terms are often used interchangeably; and there is uncertainty regarding the timeframe over which maternal morbidity impacts a woman’s health and wellbeing. Additionally, there is limited understanding of best practices to measure different components of maternal ill-health and descriptions of morbidities, and if and how different types of morbidities are interlinked and associated.

### Objective

A systematic review of the literature was conducted for studies from LMIC that measured two or more different types of maternal morbidity and/or associations with and between morbidities.

## Methods

We included studies which assessed two or more types of maternal morbidity in women during and/or after pregnancy. For the purposes of this study we categorised maternal multimorbidity as physical (such as but not limited to medical, infectious, obstetric), psychological (such as but not limited to depression, suicidal ideation) and social co-morbidities (such as but not limited to domestic violence, substance misuse) [5]. We assessed tools that were used to collect data, including self-reported subjective measures; and/or objective measures such as clinical examination; and/or use of investigations for different types of maternal multimorbidity as reported by authors. We described how and what different types of maternal multimorbidity (physical, psychological, social) were measured and if there were any reported associations between these.

### Data sources and search strategy

This protocol is registered in PROSPERO (CRD42018079526). Relevant articles published between January 2007 and December 2018 were identified using a structured search strategy in four electronic databases: CINAHL Plus, Global Health, Medline, and Web of Science. A search strategy was developed using thesaurus (including MeSH) and free-text terms for “maternal morbidity” and associated keywords, were used as main search terms. For each aspect of maternal morbidity (“physical”, “psychological” and “social”) search terms and related keywords were selected (Supplementary Table 1). Reference lists and bibliographies of key topic articles were also searched and any additional papers that met the inclusion criteria were obtained.

### Inclusion and exclusion criteria

The study population was limited to women during pregnancy, childbirth or up to 12 weeks postnatal. Studies were excluded if: (i) they reported one type of maternal morbidity only, (ii) examined trend, risk factors or associations only without estimates of prevalence of types of morbidity, or (iii) reported severe or life-threatening complications of pregnancy or childbirth that would require emergency obstetric care. The review was limited to studies from LMIC as defined by the World Bank. Language was limited to English.

### Selection and data extraction

One researcher screened all titles and abstracts (MMc). A sub-sample (20%) was double screened by the second researcher (SZ). Evaluation of full-text papers was done independently by these two researchers with reasons for exclusion recorded and any discrepancies were discussed with a third researcher (NvdB). Information was extracted into a pre-designed summary table and included data on location of study, study dates, study design, study population, types of maternal morbidity, methods of measurement, timing (pregnancy phase) of the assessment and whether or not associations were reported (Supplementary Table 2). Throughout the review and extraction process, articles where uncertainty existed were discussed by all researchers to reach consensus.

### Quality assessment

Appraisal of the quality of studies was conducted based on descriptions of maternal morbidities, sampling methods and completeness of data. Quality of evidence for each study was assessed using the Grading of Recommendations, Assessment Development and Evaluation (GRADE) tool adapted from the Critical Analysis Skills Programme (CASP) tool [14].

### Data synthesis

A narrative synthesis approach was used to describe outcomes including: types of maternal morbidity categorised as physical (such as medical, infectious, obstetric), psychological (such as depression, suicidal ideation) and social (such as domestic violence, substance misuse); approaches used to collect data (self-reported or determined by healthcare providers); data collection tools used (standardised validated tool, or study specific); measurements of maternal morbidities; and reported associations (if any) between different types of maternal morbidities.

## Results

**Fig. 1:**
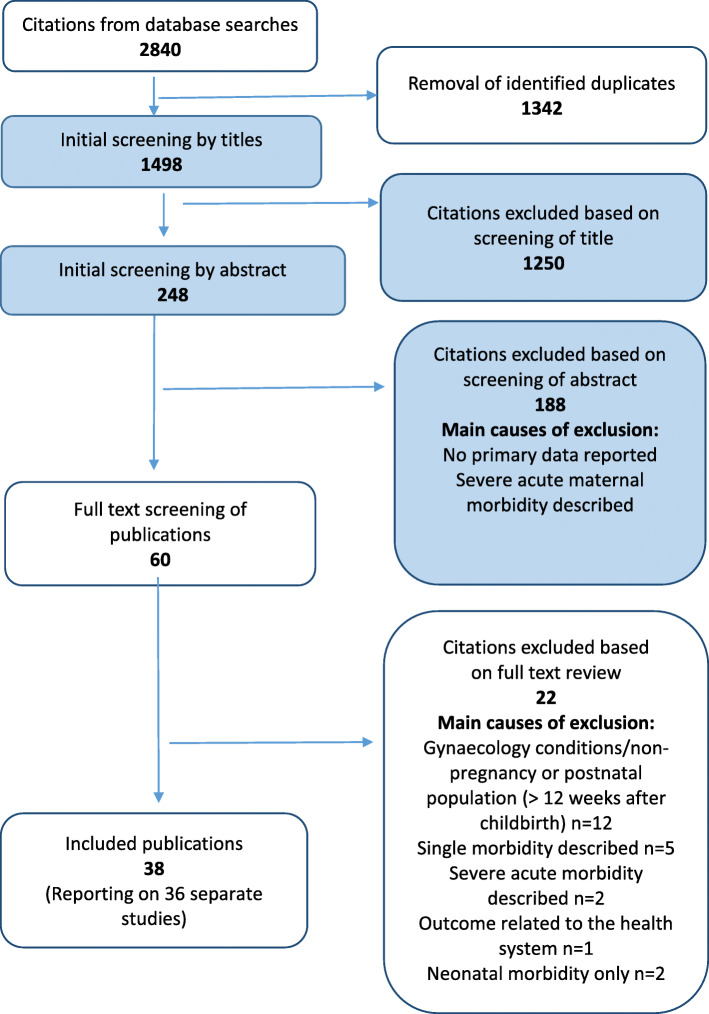
PRISMA diagram for article selection process

By combining the search terms, 2840 studies were identified from the four databases and after screening for relevance, 58 were retrieved for full text review (Fig. 1). Upon applying the eligibility criteria, 38 articles met the inclusion criteria. Two studies were conducted by the same group of authors [15–18]. In these publications, the same methodology was reported in two papers, but there was a different emphasis on the results and outcomes reported per publication. For the purposes of this review, the first publication is referenced in the methodology section [15, 17]. Both publications were included in the summary tables and measurements and/or associations for each publication are described in the results section. Most studies (92%; 33/36) were of medium quality, and the rest low quality (8%, 3/36).

### Characteristics of studies

The 36 studies were from 17 different countries, with 15 from sub-Saharan Africa. Eleven were conducted in low-income countries and six in middle-income countries (four lower-middle and two upper-middle income countries).

#### Study design, source of data, data collection method and sample size

Twenty-six studies used cross-sectional survey study designs. Four were observational prospective cohort studies [15, 19–21]. One study was a case control [22]. Twenty five studies used face-to-face interviews or consultations to collect self-reported primary data from women using questionnaires. Most studies that collected primary data relied on women’s self-reported symptoms (*n* = 28). In four studies, clinical examination and/or laboratory tests were also conducted [6, 20, 23, 24]. Three studies extracted data using secondary data analysis of large databases of hospital admissions, discharges or birth registers [25–27]. In these secondary data analyses authors used their own data collection tools with little details of the variables extracted. One study extracted data from medical case notes [28] (Supplementary Table 2). A total number of 71,229 women were assessed in the 36 studies: 60,911 during pregnancy and 10,318 after childbirth. In nine studies less than 500 women were assessed [24–33]; thirteen assessed 500–999 women each [15, 18, 20, 21, 34–43]. In nine studies 1000–1999 women were assessed [19, 22, 27, 28, 44–48]; and five had sample sizes of ≥2000 women (Supplementary Table 2) [6, 25, 26, 49, 50].

#### Stages of pregnancy assessed

A total of 23 studies collected data from women during pregnancy: in the second trimester [16, 19, 32, 40, 47]; in the third trimester [20, 24, 37]; or at any time during pregnancy [19, 29, 32, 33]. In 11 of those, gestational age was not given [32, 36, 38–45, 48, 49]. Seven studies assessed women within 12 weeks of childbirth [18, 22, 29, 30, 32, 46, 50]. In one study, data was collected during three stages after childbirth: at 4–12 weeks; at 12–24 weeks; and at 24–56 weeks [23]. Zafar et al. collected data at three different assessment stages, during early and late antenatal period and after childbirth (Supplementary Table 2) [6].

#### Site of data collection

In studies that collected primary data (*n* = 30), data collection took place during visits for routine antenatal or postnatal care in outpatient departments of healthcare facilities: tertiary/provincial hospitals [23, 31, 35, 38, 41, 44, 46]; secondary level or district hospitals [32, 43, 51], and primary healthcare facility level [15, 18, 36, 39, 47]. For four studies the site was unclear [29, 33, 34, 42]. In 12 studies, this took place in the community or in women’s homes (Supplementary Table 2) [6, 19, 20, 22, 24, 30, 42, 45, 49–51].

### Maternal multimorbidity

All three types of maternal morbidity including physical, psychological and social ill-health were assessed in 12 studies [6, 15, 16, 20, 39, 41–43, 46, 48]; psychological and social ill-health were assessed in nine studies [18, 25, 29, 31, 32, 34, 35, 39, 42]; physical and psychological ill-health in 11 [6, 18, 19, 21, 22, 24, 27, 29, 32, 33, 49]; and physical and social ill-health in six [17, 26, 44, 45, 47, 50] (Supplementary Table 2).

### Physical morbidity

Twenty-nine studies reported on different types of physical morbidity; three of which assessed pre-selected populations including women with HIV [35, 42] or women with gestational diabetes [38]. A variety of data collection tools were used, but generally not well described. No study used validated questionnaires or international disease classifications. The most commonly reported physical morbidities were anaemia in six studies (prevalence range 5.0–57.7%) [6, 20, 22, 23, 32, 49], and HIV in nine (prevalence range 3.0–16.0%) [6, 16, 29, 33, 35, 36, 43, 48, 50]. There was a variety of other types of physical morbidities, with wide ranges for some conditions such as antepartum haemorrhage; nausea and vomiting; preterm birth; malaria; reproductive or sexually transmitted infection; urinary tract infection (Supplementary Table 3). Some authors used summative aggregated measures, for example “gynaecological and obstetric problems” as occurring in 10–22% of women; “multiple morbidities” in 60% of women or “at least one reported symptom” (44% occurrence) [22, 46, 49]. One study used antenatal hospitalisation as a “proxy” for physical morbidities (55.4% of women) [45] (Supplementary Table 3).

### Psychological morbidity

Of the 32 studies that report psychological morbidities, the most common condition was depression with a prevalence range of 13.5–39.5% across 21 studies [18, 20–24, 27–35, 38, 41, 49, 51]. Twelve studies described more than one psychological condition [15, 16, 19, 25, 34, 37, 42, 43, 46–49]. Some authors described aggregates or a summative psychological condition; for example, “common mental disorders” and “symptoms of any mental distress” [15, 16]. There was a range of other types of psychological morbidity described, such as anxiety; suicidal ideation; and distress (Supplementary Table 4). Fourteen different data collection tools were used either alone or in combination (Table 1). The commonest tool was the Edinburgh Postnatal Depression Score (EPDS) questionnaire, used in fourteen studies [6, 18, 20, 28–31, 34–36, 38, 41, 49]. However, different studies used various cut-off scores (from ≥4 to ≥13) for the EPDS questionnaire and the Kessler scale (from > 15 to > 30) [6, 18, 20, 28–31, 34–36, 38, 41, 49, 54, 56].

**Table 1:** Description of data collection tools used to assess psychological and social morbidity

**No.**	**Data collection tools to assess psychological morbidity**	**International abbreviation**	**Original country, author, and date**
1.	Aga Khan University Anxiety and Depression Scale	AKUADS	Pakistan, Ali 1988 [52]
2.	Clinical Interview Schedule-Revised	CIS-R	USA, Lewis 1992 [53]
3.	Edinburgh Postnatal Depression Scale	EPDS	UK, Cox 1987 [54]
4.	Harvard Trauma Questionnaire	HTQ	USA, Mollica 1992 [55]
5.	Kessler-10 item psychological distress scale	K-10	USA, Kessler 2002 [56]
6.	List of Threatening Experiences questionnaire	LTE-Q	UK, Brugha 1985 [57]
7.	Montgomery–Åsberg Depression Rating scale	MADRA	UK, Montgomery 1979 [58]
8.	Patient Health Questionnaire	PHQ-9	USA, Spitzer 1992 [59]
9.	Self-Reporting Questionnaire-(20 Items)	SRQ-20	WHO, Switzerland, Beusenberg 1994 [60]
10.	State-Trait Anxiety Inventory	STAI	USA, Spielberger 1983 [61]
11.	Structured Clinical Interview for the Diagnostic and Statistical Manual of Mental Disorders, 4th Edition	SCI- DSM IV	USA, American Psychiatric Association 1994 [62]
12.	WHO version of the Centre for Epidemiological Studies Depression scale	CES-DR	USA, Radloff 1977 [63]
13.	Mini-intentional neuropsychological	MINI	USA, Sheehan, 1998 [64]
**No.**	**Data collection tools to assess social morbidity**	**International abbreviation**	**Original country, author, and date**
1.	Alcohol Use Disorders Identification Test	AUDIT	WHO, Babor 2001 [65]
2.	CAGE (Cut-annoyed-guilty-eye) Questionnaire	CAGE	USA, Ewing 1970 [66]
3.	Maternity Social Support Index	MSSI	USA, Pascoe 1988 [67]
4.	Social Provisions Scale	SPS	USA, Cutrona 1987 [68]
5.	HIV-AIDS Stigma Instrumental PHWHA	HASI-P	WHO, Babor 2001 [69]

### Social morbidity

In total, 27 studies assessed social morbidity; the most commonly reported type of social morbidity was domestic violence in 14 studies [17, 18, 23, 25, 28, 31, 35, 38, 39, 43, 45, 47, 48, 50]. Substance abuse was assessed in nine studies (Supplementary Table 4) [16, 34, 39, 40, 42, 44–46, 50]. Three studies assessed both domestic violence and substance abuse [26, 37, 46]. Eight studies assessed other aspects of social health including husband’s alcohol intake, poor social support, food insecurity and unplanned pregnancy [20, 21, 30, 36, 41, 42, 46, 51].

In the 14 studies assessing domestic violence, a variety of data collection tools were used and most authors used their own definitions and questionnaires to screen for domestic violence. Five publications used all or part of internationally recognised questionnaires (Table 1) [17, 18, 39, 45, 47]. Different types of domestic violence included: disrespect, forced sex, intimate partner violence, physical assault, severe emotional and verbal abuse. Other authors used descriptions of domestic violence as aggregates or summative measures, for example, terms such as “multiple acts of physical violence” and “physical and/or sexual abuse” [38]. Nine studies assessed one or more forms of substance abuse [16, 34, 39, 40, 42, 44–46, 50], and two of these used validated questionnaires [34, 40]. In general, substance abuse related to alcohol (9 studies; prevalence range 0–49.5%) [16, 34, 39–41, 44–46, 50].

### Associations between different types of morbidity

For physical morbidity, there was an association between increased psychological morbidity in women with obstetric complications (haemorrhage, infections, incontinence, prolonged labour, caesarean birth, low birthweight, stillbirth, neonatal death) (Table 2) [6, 26, 41, 45, 50]. Women with gestational diabetes were not more likely to have psychological morbidity (depression) [38], but women with HIV were more likely to have social morbidity (domestic violence) [18]. Psychological morbidity was more common in younger women [40] and among women with social morbidities such as domestic violence [25, 35], unwanted pregnancy [19, 41, 50] and poor social support (Table 2) [41]. For social morbidity, there was an association between women with substance abuse (alcohol) and domestic violence [48]^;^ and domestic violence was also associated with neonatal death [48] and maternal complications (Table 2) [46]. Due to heterogeneity, meta-analysis of associations was not possible.

**Table 2:** Associations between types of maternal morbidity

Type of morbidity	Author, date	Associations between different types of maternal morbidity
**Physical morbidity**	Shamu 2014 [18]	Positive HIV status was associated with intimate partner violence for pregnant women: partially adjusted OR 1.43: (95%CI: 1.00–2.05).
	Surkan 2017 [26]	In models adjusted for sociodemographic factors and co-morbidities, all postpartum illnesses were associated with an increased relative risk of depressive symptoms in women by 6 months postpartum. These morbidities included uterine prolapse (RR 1.20, 95% CI 1.04–1.39), urinary tract infection (RR 1.24, 95% CI 1.11–1.38), stress related incontinence (RR 1.49, 95% 1.33–1.67), simultaneous stress related incontinence and continuously dripping urine (RR 1.60–2.96), headache [RR 1.20 (95% CI 1.12–1.28)], convulsions (RR 1.67, 95%CI 1.36–2.06), night blindness (RR 1.33, 95% CI 1.19–1.49), anaemia (RR 1.38, 95% CI 1.31–1.46), pneumonia (RR 1.24, 95% CI 1.12–1.37), gastroenteritis (RR 1.24, 95% CI 1.17–1.31) and hepatobiliary disease (RR 2.10, 96% CI 1.69–2.60).
	Zafar 2015 [6]	Multivariate logistic regression showed that for pregnant women in Malawi, after controlling for parity and pregnancy stage, antepartum bleeding increased the odds of psychological morbidity 5-fold (OR: 5.01; 95% CI 1.60, 15.70; *p* = 0.006). Infective morbidity (i.e. for each additional infective morbidity) showed more than 2.5-fold increase in the odds of having psychological morbidity (OR: 2.58; 95% CI 1.92, 3.47; *p* = 0.000). For Pakistan, there was a 56% increase in odds of psychological morbidity due to increasing burden of infective morbidity (OR: 1.56; 95% CI 1.36, 1.79; *p* = 0.000), and 78% increased odds due to increasing burden of non-infective morbidity (OR: 1.78; 95% CI 1.51, 2.11; p = 0.000), when controlling for the effect of complications during previous pregnancy. Complications during previous pregnancy, infective morbidity (*p* < 0.001), intra or postpartum haemorrhage (*p* < 0.02) were associated with psychological morbidity in both settings.
**Psychological morbidity**	Faisal-Cury 2009 [16]	Obstetric complications were independently associated with common mental disorders in pregnant women.
	Faisal-Cury 2010 [15]	Common mental disorders during pregnancy were not associated with risk of preterm birth (adjusted OR: 1.03, 95% CI: 0.57–1.88) or low birth weight (adjusted OR: 1.09, 95% CI: 0.62–1.91).
	Karmaliana 2009 [19]	Psychological distress in pregnant women was associated with husband unemployment (*p* = 0.032), lower household wealth (*p* = 0.027), having 10 or more years of formal education (*p* = 0.002), first (p = 0.002) and unwanted pregnancies (p < 0.001).
	Hanlon 2009 [45]	Significant associations exist between pregnant women who report intimate partner violence and preterm labour, need for caesarean section, antenatal hospitalization and vaginal bleeding.
	Nasreen 2011 [37]	Increasing levels of common mental disorder symptoms in pregnant women were associated with prolonged labour (> 24 h) (SRQ 1–5: RR 1.4; 95% CI 1.0–1.9, SRQ > or = 6: RR 1.6; 95% CI 1.0–2.6).
	Natasha 2015 [38]	There was no association between women with depression and gestational diabetes mellitus or other obstetric factors. However, pregnant women’s level of literacy, poor household economy, poor relationship with husbands, and partner violence showed strong associations with depression and anxiety.
	Prost 2012 [50]	Unwanted pregnancy, small perceived infant size and stillbirth or neonatal deaths were all independently associated with increased risk of psychological distress in postnatal women. Loss of infants or unwanted pregnancies increased the risk of distress considerably (aORs: 7.06 95% CI: 5.51–9.04 and 1.49, 95% CI: 1.12–1.97).
	Rees 2016 [47]	For pregnant women with any mental distress, adjusted odds ratios for four or more traumatic events and severe psychological abuse was 3.60 (95% CI 2.08–6.23); for four or more traumatic events and physical abuse 7.03 (95% CI 3.23–15.29); and for four or more traumatic events and severe psychological and physical abuse the adjusted OR was 10.45 (95% CI 6.06–18.01). For pregnant women who reported four or more traumatic events, and either physical abuse alone or in combination with severe psychological abuse, there was a 10-fold increase in depressive and other mental health symptoms.
	Ukacukw 2009 [28]	After multivariable adjustment, intimate partner violence intensity had a strong and statistically significant association with depression symptom severity for pregnant women.
	Waqas 2015 [41]	Results of unadjusted log-binomial regression showed that unwanted pregnancy, prenatal depression and social support were associated with low birth weight.
	Wong 2017 [42]	Inferential analysis revealed that higher HADS scores were significantly associated with lower social support scores, rural background, history of harassment, abortion, caesarean birth and unplanned pregnancies (*P* < .05).
**Social morbidity**	Hassan 2014 [44]	A significant association was found between pregnant women reporting intimate partner violence and preterm labour [adjusted odds ratio (adjOR) 1.54, 95% confidence interval (CI) 1.16–2.03], caesarean section (adjOR 11.84, 95% CI 6.37–22.02), antenatal hospitalization (adjOR 6.34, 95% CI 3.82–10.52) and vaginal bleeding (adjOR 1.51, 95% CI 0.9–2.3).
	Romero-Gutiérrez 2011 [46]	Maternal complications were higher in pregnant women who experienced violence (30.2% vs 23.6%, *p* = 0.004). Pregnant women who experienced sexual violence had more maternal complications (43.2%), and pregnant women who experienced psychological violence had more neonatal complications (54.2%).
	Stöckl 2010 [48]	Women’s odds of drinking alcohol during pregnancy were significantly increased if they had experienced violence during pregnancy. Violence during pregnancy was also associated with having had a child or infant that died.

## Discussion

### Main findings

There is emerging evidence of a high burden of multimorbidity in women living in LMIC during pregnancy and after childbirth, as well as emerging evidence of associations between physical, psychological and social morbidities, suggesting that maternal morbidities are inter-linked. There is, however, still limited data about the strengths and direction of the associations between the different types of morbidities.

There was an apparent lack of standardisation of definitions and data collection tools used to measure maternal multimorbidities. The EPDS was the most common validated data collection tool to assess psychological morbidity in the studies, but with different cut-off scores to determine the risk of “depression” (ranging from 4 to 13) making comparisons difficult. Similarly, a variety of different validated data collection tools were used to assess domestic violence and/or substance abuse as components of social morbidity. Physical, psychological and social morbidities were often described as aggregates or summative measures, limiting comparability of findings.

### Strengths and limitations

To the best of our knowledge, this is the first systematic review to assess maternal multimorbidities and types and levels of association between the different types. Many studies relied on recall of experience of morbidity and many primary data were symptom-based rather than “diagnosed”. Only four studies triangulated self-reported symptoms with findings from clinical examination and/or basic laboratory investigations. Assessments of measurements of ill-health based on self-reporting may be valid regarding ill-health as experienced by women, but do not provide accurate burden of disease estimates. No study described or used internationally recognised disease classifications to assess physical morbidity. Internationally recognised data collection tools were used to assess psychological and social morbidity, but these often used different cut-off scores making comparisons difficult. A limitation of this review is that studies that explored maternal multimorbidity using qualitative methodology were excluded.

### Interpretation

Valid comparable measurements of maternal multimorbidity are limited to date, and this study confirms the need for a new approach and focus [70–72]. It will be important for future healthcare practice and research to agree and apply: (a) common identification criteria for maternal multimorbidity taking into account the different types of physical, psychological and social morbidity; (b) standardised and validated data collection tools that can be used in different languages and at all levels of healthcare; with, (c) validation of self-reported measurements of maternal morbidity compared to clinical assessment, investigations and diagnosis determined by healthcare providers [6, 70–72]. More recognition must be given that maternal morbidity is a complex concept with important associations between different morbidities. This has implications for screening and management of all different types of ill-health during pregnancy and after childbirth. There is a need to incorporate women’s understanding, perceptions and lived experience of maternal multimorbidity into public health approaches to improve maternal health and wellbeing during pregnancy and after childbirth in LMIC [73, 74].

## Conclusion

To date a range of methods and tools have been used to assess maternal multimorbidity. Maternal multimorbidity estimates using these methodologies and tools, while useful as a guide, cannot be considered truly representative of the burden and range of maternal multimorbidity that have negative impact on women’s wellbeing during pregnancy and after childbirth. The suggested WHO definition of maternal morbidity provides such a framework in principle, but challenges remain to map out comprehensive, feasible and acceptable assessment tools, approaches and timeframes [11]. Comprehensive and routine measurements of maternal multimorbidity are necessary to inform policy and program decisions and for resource allocation for antenatal and postnatal care [5]. Improved standardised measurements of maternal multimorbidity will also allow for comparison of the burden of disease across settings within and between countries. There is a need for a sustainable way to provide good baseline maternity care for all and targeted individualised care for women who need extra care to prevent development and progression of maternal multimorbidity.

## Supplementary information

**Additional file 1: Supplementary Table 1.** MeSH terms and keywords used in the search.

**Additional file 2: Supplementary Table 2.** Summary table for studies reporting maternal multimorbidity during pregnancy and after childbirth and /or associations between the multimorbidities.

**Additional file 3: Supplementary Table 3.** Summary table of types of physical morbidity reported in included studies.

**Additional file 4: Supplementary Table 4.** Summary table of measurements of psychological and social morbidities reported.

## Data Availability

All the sources of data are publicly available and referenced in the document.

## Abbreviations

EPDS: Edinburgh Postnatal Depression Score
CASP: Critical Analysis Skills Programme
GRADE: Grading of Recommendations, Assessment, Development and Evaluation
LMIC: Low- and middle-income countries
MeSH: Medical Subject Headings
PRISMA: Preferred Reporting Items for Systematic Reviews and Meta-Analyses
WHO: World Health Organization
