# Supplementary material for: Maternal multimorbidity during pregnancy and after childbirth in women in low- and middle-income countries: a systematic literature review
Source: BMC Pregnancy Childbirth. 2020 Oct 20;20:637. doi: 10.1186/s12884-020-03303-1 (PMC7574312; doi:10.1186/s12884-020-03303-1)
Supplement: Supplementary file 1 — Additional file 1: Supplementary Table 1. MeSH terms and keywords used in the search. [file 12884_2020_3303_MOESM1_ESM.docx]

## Supplementary Table 1: MeSH terms and keywords in the search

| **Topic** | **MeSH terms** | **Keywords** |
| --- | --- | --- |
| **Maternal** | Parturition OR  Pregnancy* OR  Prenatal care OR  Postnatal care OR  Obstetric labor complication* | Labor OR Obstetric OR Puerperal OR  Maternal OR Delivery OR Intrapartum OR Antenatal |
| **Morbidity** | Morbid* OR  Pregnancy complications | Unwell OR Ill* OR Disorder* OR Disease* |
| **Physical** | Infection* OR  Puerperal infection* OR  Sepsis OR Systematic inflammatory response syndrome* OR HIV* OR  Malaria OR tuberculosis OR syphilis OR  Haemorrhage OR haemorrhage OR Postpartum haemorrhage OR urinary incontinence OR  Anemia or anaemia OR  Pre-eclampsia OR  Hypertension OR  Pregnancy induced hypertension | Medical OR  Obstetric |
| **Psychological** | Mental health OR Depression OR Postpartum depression OR  Self-mutilation OR  Suicide OR Anxiety OR Psycho* OR Neurosis OR Mental disorder* OR  Stress disorder* | Suicidal ideation OR  Self-harm |
| **Social** | Domestic violence OR  Intimate partner violence OR  Substance related disorders OR  Alcohol* OR Tobacco use OR  Smoking OR Street drugs OR  Inhalant abuse OR  Hypnotics and sedatives | Gender based violence OR  Domestic abuse |
| **Low resource setting** | Developing countr* OR  Low income countr* OR  Middle income countr* OR  Low resource setting* OR  Global south |  |
| **Limitations** | Human  Female  2007-2018 |  |
