# Supplementary material for: Maternal multimorbidity during pregnancy and after childbirth in women in low- and middle-income countries: a systematic literature review
Source: BMC Pregnancy Childbirth. 2020 Oct 20;20:637. doi: 10.1186/s12884-020-03303-1 (PMC7574312; doi:10.1186/s12884-020-03303-1)
Supplement: Supplementary file 3 — Additional file 3: Supplementary Table 3. Summary table of types of physical morbidity reported in included studies. [file 12884_2020_3303_MOESM3_ESM.docx]

## Supplementary Table 3: Summary table of types of physical morbidity

| **Medical/obstetric morbidity** | **Prevalence or range (%)** | **Infectious morbidity** | **Prevalence or range (%)** |
| --- | --- | --- | --- |
| Abdominal pain | 17.0 | Fever / febrile symptoms | 2.4 - 13.1 |
| Abortion / miscarriage | 1.0 - 22.0 | Gastroenteritis | 22.1 |
| Anaemia | 5.0 - 57.7 | Hepatitis | 1.6 |
| Antepartum haemorrhage | 3.1 - 31.0 | HIV positive | 3.0 - 16.0* |
| Asthma | 1.0 - 1.4 | Malaria | 2.7 - 15.9 |
| Breast problems | 5.0 | Pneumonia | 4.9 |
| Epilepsy | 0.3 - 0.8 | Reproductive tract infection | 2.5 – 31.0 |
| Episiotomy problems | 16.2 | Sexually transmitted infection | 7.0 - 14.9 |
| Genital tract trauma | 90.6 | Tuberculosis (suspected) | 0.8 - 10.0 |
| Gestational diabetes | 51.0* | Urinary tract infection | 2.0 - 14.5 |
| Hypertension | 13.0 |  | |
| Incontinence | 0.33 - 4.7 |  |  |
| Maternal malnutrition | 68.1 |  |  |
| Nausea and vomiting | 18.8 - 57.7 |  |  |
| Pre-eclampsia | 0.2 - 0.8 |  |  |
| Premature rupture of the membranes | 0.1 |  |  |
| Preterm birth | 6.9 |  |  |
| Preterm labour | 30.3 |  |  |
| Prolapse | 2.2 |  |  |
| Severe headache | 14.2 |  |  |

*in pre-selected populations of women with the condition of interest.
