# Supplementary material for: Maternal multimorbidity during pregnancy and after childbirth in women in low- and middle-income countries: a systematic literature review
Source: BMC Pregnancy Childbirth. 2020 Oct 20;20:637. doi: 10.1186/s12884-020-03303-1 (PMC7574312; doi:10.1186/s12884-020-03303-1)
Supplement: Supplementary file 4 — Additional file 4: Supplementary Table 4. Summary table of measurements of psychological and social morbidities reported. [file 12884_2020_3303_MOESM4_ESM.docx]

## Supplementary Table 4: Summary table of measurements of psychological and social morbidities

| **Psychological morbidity**  **(author description in alphabetical order)** | | **Range of prevalence**  **(%)** | **Social morbidity**  **(author description in alphabetical order)** | | **Range of prevalence (%)** |
| --- | --- | --- | --- | --- | --- |
| **Anxiety** | Anxiety | 29.0 | **Domestic violence** | |  |
| **Depression** | “Depression” | 13.5 - 39.5 | **One form of domestic violence** | Disrespect | 32.9 |
| **Depression**  **Suicidal ideation** | EDPS score ≥ 4 | 16.9 |  | Forced sex | 79.2 |
|  | EPDS score >9 | 2.6 - 26.9 |  | Intimate partner violence | 13.0 - 35.4 |
|  | EPDS score ≥10 | 18.0 - 19.0 |  | Physical assault | 2.3 - 30.2 |
|  | EPDS score ≥12 | 9.2 |  | Severe psychological abuse | 30.6 |
|  | EPDS score ≥13 | 11.0 - 19.7 |  | Verbal abuse | 30.0 |
|  | Minor depression | 10.4 |  | Violence  (no details of type) | 8.8 - 43.8 |
|  | Major depression | 2.0 - 10.7 |  | At least one form of physical, emotional or sexual violence | 60.0 - 72.0 |
|  | Postpartum suicide thoughts | 21.6 | **More than one form of domestic violence** | Multiple acts of physical violence | 33.8 |
| **Suicidal ideation**  **Distress** | “Suicidal ideation” | 7.6 - 29.0 |  | Physical and/or sexual abuse | 15.0 |
|  | Attempted suicide | 4.0 |  | Physical and psychological abuse | 19.5 |
|  | Distress (Kessler score >15) | 11.5 - 33.0 |  | Fear of family members | 5.9 |
| **Distress**  **Stress** | Distress (Kessler score >30) | 6.3 | **Substance misuse** | | |
|  | Post-traumatic stress disorder score ≥2 | 5.7 | **Alcohol** | Alcohol use | 0.0 - 49.5 |
|  | High symptom levels (SRQ scores ≥6) | 12.0 |  | Alcohol-related harm | 21.0 |
| **Stress** | Low symptoms  (SRQ scores 1–5) | 59.5 |  | Risky alcohol use | 15.7 - 16.0 |
|  | No symptoms (SRQ = 0) | 28.5 | **Stimulant** | “Khat” (stimulant) | 12.9 |
|  | Anxiety and/or depression | 18.0 | **Tobacco** | Smoker | 52.6 |
